# Evidence that the p53 negative / Bcl-2 positive phenotype is an independent indicator of good prognosis in colorectal cancer: A tissue microarray study of 460 patients

**DOI:** 10.1186/1477-7819-3-47

**Published:** 2005-07-19

**Authors:** Nicholas FS Watson, Zahra Madjd, Duncan Scrimegour, Ian Spendlove, Ian O Ellis, John H Scholefield, Lindy G Durrant

**Affiliations:** 1 Academic Department of Clinical Oncology, University of Nottingham, City Hospital, Nottingham, NG5 1PB, UK; 2 Section of Gastrointestinal Surgery, University of Nottingham, Queens' Medical Centre, Nottingham, NG7 2UH, UK; 3 Department of Pathology, City Hospital, Nottingham, NG5 1PB, UK

## Abstract

**Background:**

Advances in our understanding of the molecular biology of colorectal cancer have fuelled the search for novel molecular prognostic markers to complement existing staging systems. Markers assessed in combination may perform better than those considered individually. Using high-throughput tissue microarray technology, we describe the prognostic value of combined p53 / Bcl-2 status in colorectal cancer.

**Patients and methods:**

Tumour samples from 462 patients who underwent elective surgery to resect a primary colorectal cancer between 1994 and 2000 (mean follow-up of 75 months) were assembled in tissue microarray format. Clinico-pathological data including tumour grade, stage, vascular invasion status along with disease specific survival data has been collected prospectively. Immunohistochemical analysis of p53 and Bcl-2 expression was performed using antibodies DO-7 (p53) and 124 (Bcl-2), and results correlated with known clinico-pathological variables and outcomes.

**Results:**

Abnormal nuclear p53 accumulation and Bcl-2 overexpression were detected in 221/445 (49.6%) and199/437 (45.5%) tumours respectively, with a significant inverse correlation between the two markers (p = 0.023). On univariate analysis no correlations were found between either marker and standard clinico-pathological variables, however nuclear p53 expression was associated with a significantly reduced survival (p = 0.024). Combined analysis of the two markers indicated that 112/432 (24.2%) cases displayed a p53(-)/Bcl-2(+) phenotype, this occurring more frequently in earlier stage tumours. Kaplan-Meier analysis revealed a significant survival advantage in these p53(-)/Bcl-2(+) tumours compared with the remaining cases (p = 0.0032). On multivariate analysis using the Cox proportional hazards model, neither p53 expression nor Bcl-2 expression alone were of independent prognostic significance, however the combined p53(-)/Bcl-2(+) phenotype was significantly associated with a good prognosis in this series (HR 0.659, 95%CI 0.452–0.959, p = 0.029).

**Conclusion:**

Patient stratification by combined p53 / Bcl-2 phenotype provides stage-independent prognostic information in colorectal cancer. Specifically, that up to a quarter of patients display a good prognosis p53(-)/Bcl-2(+) phenotype. This may indicate a more clinically indolent phenotype and a subset of patients for whom less aggressive adjuvant treatment appropriate.

## Background

Colorectal cancer kills approximately 500 000 people worldwide each year [1], thus treatments which produce only modest improvements in survival may have an enormous health impact. Current practice is to base therapeutic decisions and prognostic advice on clinico-pathological data, however within conventional staging groups many genetic and molecular tumour subtypes exist. This heterogenicity of tumour genotypes accounts for much of the observed variation in recurrence rates and clinical responses to available therapies.

Advances in our understanding of the molecular biology of colorectal cancer have fuelled the search for novel molecular prognostic markers with which to complement existing staging systems. Applied to clinical practice, these putative markers could be used to identify groups of patients with differing relative risks of recurrence and improve patient stratification for adjuvant treatment.

Immunohistochemical studies in colorectal cancer have tended to investigate expression of individual proteins in relation to prognosis, and relatively few studies have focused on the analysis of multiple markers in combination. However, one such combination which may be of value in colorectal cancer is the combined p53 / Bcl-2 phenotype, as suggested by Manne *et al*, who described the p53/Bcl-2 phenotype of 134 patients with Dukes stage A-D tumours, finding that this combination gave independent prognostic information which was superior to that of either marker on its own [2]. The same group have subsequently shown in a larger cohort of 234 patients with tumours of the distal colo-rectum that combined p53/Bcl-2 analysis may provide stronger prognostic information than nodal status [3]. Other researchers have reported similar findings in differing groups of colorectal cancer patients [4,5], with the p53(-)/Bcl-2(+) subset appearing to define a group of patients who appear to have a prolonged survival, although this has not always retained independence on multivariate analysis [6,7]. Alternatively, it has been suggested that opposing p53(+)/Bcl-2(-) phenotype defines a particularly poor prognosis subset [8], or that Bcl-2 positivity in combination with either p53, p21 or mdm-2 confers a good prognosis [9].

Since its first description in 1998, tissue microarray (TMA) analysis [10] has been employed for the immunohistochemical analysis of target protein expression in a wide range of primary tumour types. Initial fears that the reduced amount of individual tumour tissue analysed using this technique might not be representative of the tumour as a whole appear largely unfounded [11], and the strengths of this approach lie in its ability to provide a rapid turnover of results from very large patient cohorts, whilst reducing variability in experimental conditions and reducing costs [12]. Two previous studies have utilised tissue microarray technology to investigate the combination of p53/Bcl-2 in rectal cancer. In the first, Hoos *et al*, studied 100 patients with lymph-node negative rectal cancer, and found an increased number of deaths in the patients with p53(+)/Bcl-2(-) tumors (7/33, 21.2%) compared with the p53(-)/Bcl-2(+) group (2/13, 15.4%) [13], and a recent study of 269 patients with Dukes stage A-D rectal cancer reported a lower rate of metastatic disease in p53(-)/Bcl-2(+) tumours, with p53(+)/Bcl-2(-) tumours showing a worse outcome [14].

The aim of the present study was to investigate the prognostic significance of the combined p53/Bcl-2 phenotype in a large scale, representative sample of colorectal cancers using tissue microarrays, in order to determine the potential utility of this combination of molecular markers in guiding treatment of patients in the UK.

## Patients and methods

### Patients and specimens

The study population comprised 462 patients undergoing elective surgery for a single, non-metachronous histologically proven primary colorectal cancer at University Hospital Nottingham between 1^st ^January 1994 and 31^st ^December 2000. Data regarding tumour site, stage, histological type and tumour grade have been recorded prospectively for these patients. Only patients with lymph node positive disease were routinely treated with adjuvant chemotherapy, comprising 5-flurouracil and folinic acid. The original histopathological slide sets and pathological reports for all cases were obtained from the hospital archives, and reviewed to confirm the diagnosis and the accuracy of existing data. However, in a minority of cases only very limited slide sets were available and the full clinico-pathological dataset was incomplete. In addition, we did not attempt to ascertain the vascular invasion status of tumours where this information had not been previously recorded.

Follow-up data regarding the date and cause of death for this cohort of patients has been provided prospectively by the UK Office for National Statistics. Follow-up was calculated from the date of resection of the primary tumour, and all surviving cases were censored for survival analysis at 31^st ^December 2003. Disease specific survival was used as the primary end-point. The Local Research Ethics Committee granted approval for the study.

### Preparation of the tissue microarray

Tissue microarrays were constructed as described previously [10]. 5 μm haematoxylin and eosin (H&E) stained slides were used to identify and mark out representative areas of viable tumour tissue. 0.6 mm needle core-biopsies from the relevant areas of corresponding paraffin-embedded blocks were then placed at defined coordinates in the recipient paraffin array blocks using a manual arrayer (Beecher Instruments, Sun Prarie, WI). Array blocks were constructed at a density of 80–150 cores per array. Analysis of each marker was performed on a single core from each tumour. This typically shows over 90% concordance with conventional whole section analysis of tumour markers and has been validated previously [11].

### Immunohistochemical methods

Immunohistochemistry was performed using a standard avidin-biotin peroxidase method with p53 mAb clone DO-7 (1:20 dilution, Dako Ltd, Ely, UK), and Bcl-2 mAb clone 124 (1:50, Dako Ltd, Ely, UK). Briefly, 5 μm array sections were deparaffinised with xylene, rehydrated through graded alcohol and immersed in methanol containing 0.3% hydrogen peroxide for 10 minutes to block endogenous peroxidase activity. Heat induced epitope retrieval consisting of 20 minutes microwave treatment in pH 6.0 citrate buffer was necessary with the DO-7 mAb. Endogenous avidin/biotin binding was blocked using an avidin/biotin blocking kit (Vector Labs, USA) and sections were treated with 100 μl of normal swine serum (NSS) for 10 min to block non-specific binding of the primary antibody.

Test sections were incubated with 100 μl of primary antibody for 1 hr at room temperature. Positive control tissue comprised whole sections of human tonsil. Primary antibody was omitted from negative control sections, which were incubated in NSS. After washing with TBS sections were incubated with 100 μl of biotinylated goat anti-mouse/rabbit immunoglobulin (Dako Ltd, Ely, UK) diluted 1:100 in NSS for 30 min, followed by 100 μl of pre-formed streptavidin-biotin/horseradish peroxidase (HRP) complex (Dako Ltd, Ely, UK) for 60 min at room temperature. Staining was finally visualised using 3, 3'-Diaminobenzidine tetrahydrochloride (DAB, Dako Ltd, Ely, UK).

### Evaluation of staining

Evaluation of the staining was carried out by two observers (NFSW and ZM) blinded to the clinico-pathological data, with a consensus decision in all cases. For each antigen tumours were classified in two groups with the defined cut-off values of: p53 overexpression if >10% tumour nuclei stained, irrespective of staining intensity, and Bcl-2 overexpression if cytoplasmic staining (any intensity) was identified in >30% of the tumour cells. These values were determined from previous studies using the same reagents in colorectal cancer [13], although it has recently been shown in a meta-analysis that when using the DO-7 antibody, reducing the cut-off value to 1% of the nuclei stained does not alter the outcome of subsequent survival analysis [15]. In order to further investigate the significance of the combined p53 / Bcl-2 phenotypes, the individual staining patterns for these antigens were combined and then analysed as an additional group.

### Statistical methods

All statistical analyses were performed using the SPSS package (version 11 for Windows, SPSS Inc., Chicago, IL). Associations between categorical variables were examined using cross tabulation and the Pearson chi-square test for categorical variables. Kaplan-Meier curves were derived in order to assess disease-specific survival, and the significance of differences in disease-specific survival between groups was calculated using the log-rank test. Complete outcome data was available for all but one patient in the study. Patients whose deaths related to their colorectal cancer were considered in the disease-specific survival calculations. However, patients who died from postoperative complications (deaths within 1 month of the date of surgery), and patients with carcinoma in-situ (pT_is _tumors) were excluded from the survival analyses. Deaths resulting from non-colorectal cancer related causes were censored at the time of death. Multivariate analysis using the Cox proportional-hazards model was employed to determine hazard ratios and identify variables with independent prognostic significance in this cohort. In all cases *P *values <0.05 were considered statistically significant.

## Results

### Clinico-pathological data

The arrayed tumours were found to be broadly representative of the spectrum of colorectal cancer encountered in the UK (Table 1). The median age at the time of surgery was 72 years, consistent with a median age at diagnosis of colorectal cancer of 70–74 years in the UK [16]. At the time of censoring for data analysis 49% of patients had died from their disease, 37% were still alive and 14% were deceased from non-colorectal cancer related causes. 38 deaths occurred within one month of surgery. The median length of follow-up available for surviving patients was 75 months (range 36–116). The overall median five-year disease-specific survival for the cohort was 58 months, which is comparable with the approximately 45% five-year survival seen for colorectal cancer in the UK [17].

**Table 1:** Clinico-pathological variables for the patient cohort (n = 462).

**Age (years)**	Median	72
	Range	57–89
**Gender**	Male	266 (58%)
	Female	196 (42%)

**Status**	Alive	169 (37%)
	Dead (colorectal cancer related)	228 (49%)
	Dead (non-colorectal cancer causes)	64 (14%)
	Unknown	1

**Histological type**	Adenocarcinoma	392 (85%)
	Mucinous carcinoma	51 (11%)
	Columnar carcinoma	4 (1%)
	Signet ring carcinoma	7 (1%)
	Unknown	8 (2%)

**Histological grade**	Well differentiated	29 (6%)
	Moderately differentiated	353 (77%)
	Poorly differentiated	71 (15%)
	Unknown	9 (2%)

**Tumour site**	Colon	238 (52%)
	Rectal	181 (39%)
	Unknown	43 (9%)

**TNM stage**	0 (T_is_)	3 (1%)
	1	69 (15%)
	2	174 (38%)
	3	155 (33%)
	4	54 (12%)
	Unknown	7 (2%)

**Extramural vascular invasion**	Negative	224 (48%)
	Positive	128 (28%)
	Unknown	110 (24%)

### Antigen expression

**Figure 1:**
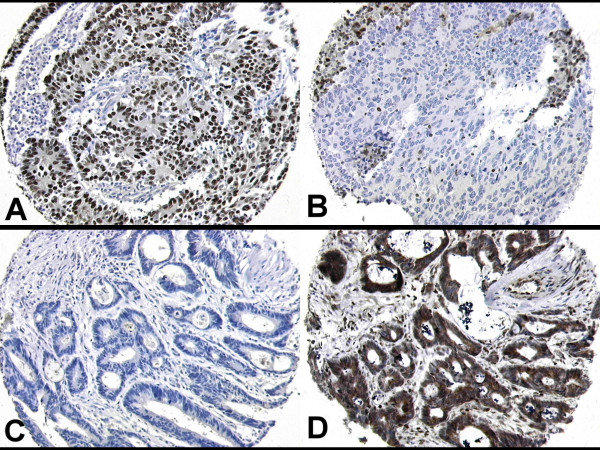
A & B show cores from a tumour demonstrating positive staining for p53 (A), and negative staining for Bcl-2 (B). C & D show cores from a p53 negative (C) / Bcl-2 positive (D) tumour. All at x20 original magnification.

The observed frequencies of expression for p53 and Bcl-2 are shown in table 2. Although it has previously been stated that the number of cores uninterpretable due to tissue loss or damage in tissue microarray studies may exceed 20–30% of the total, this was not observed in the current study. The highest number of uninterpretable cases (30 cases / 6.5% of total) was seen when the results for p53 and Bcl-2 were combined. Representative examples of positive and negative staining for each antigen are shown in figure 1.

**Table 2:** Frequencies of immunohistochemical expression of p53 and Bcl-2.

Antigen	Number (%) positive (high)	Number (%) negative (low)	Number (%) missing
p53	221 (47.8)	224 (48.5)	17 (3.7)
Bcl-2	199 (43.1)	238 (51.5)	25 (5.4)
p53 (-) / Bcl-2 (+) combination	112 (24.2)	320 (69.3)	30 (6.5)

### p53 expression

**Figure 2:**
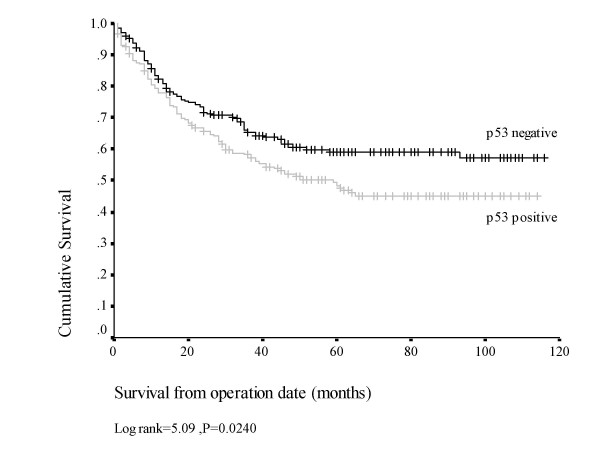
Kaplan-Meier plot for disease specific survival, p53 (+) vs p53 (-) tumours (n = 408).

p53 in its wild-type form is undetectable by conventional immunohistochemical methods, due to its short half-life. In contrast, most mutations in the p53 gene lead to expression of a stable protein product which accumulates in the nucleus and is detected by the DO-7 antibody. In this study overexpression of p53 was detected in 221/445 tumours (49.6%). On univariate analysis no associations were found between p53 expression and clinico-pathological variables including tumour grade, stage and the presence of extramural vascular invasion, however a significant relationship was noted between p53 expression and disease specific survival. On Kaplan-Meier analysis (figure 2), patients with p53(-) tumours demonstrated a significantly longer mean disease specific survival (DSS) of 76 (95% CI 69–83) months, as compared with a mean DSS of 64 (95% CI 57–71) months in patients with p53(+) tumours (p = 0.0240).

### Bcl-2 expression

**Figure 3:**
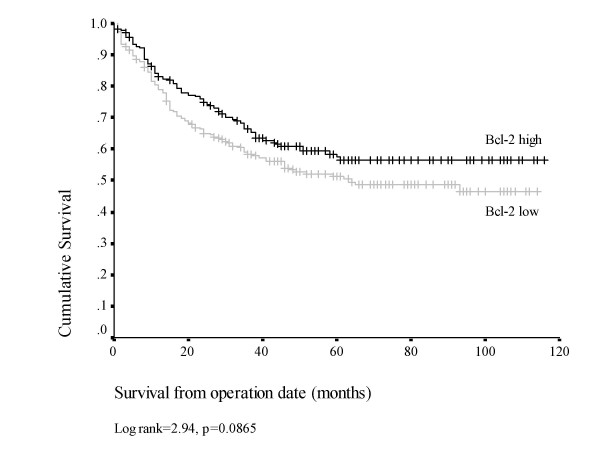
Kaplan-Meier plot for disease specific survival, Bcl-2 (+) vs Bcl-2 (-) tumours (n = 400).

Overexpression of Bcl-2 protein was detected in the cytoplasm of 199/437 evaluable tumours (45.5%). In contrast, no nuclear expression of Bcl-2 was observed. Although no strong associations between Bcl-2 expression and clinico-pathological variables were noted, there was a trend on univariate analysis towards increased cytoplasmic accumulation of Bcl-2 in moderate and poorly differentiated tumours compared with well differentiated tumours (p = 0.076). On Kaplan-Meier analysis, no association was found between Bcl-2 expression and survival (p = 0.0865, figure 3).

### p53(-) / Bcl-2(+) phenotype

**Figure 4:**
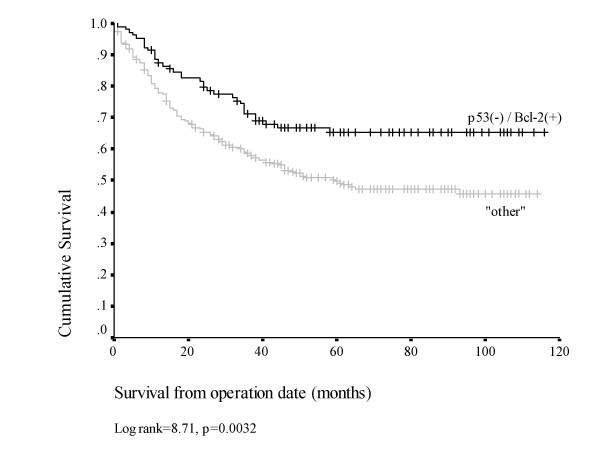
Kaplan-Meier plot for disease specific survival comparing p53 (-) / Bcl-2 (+) tumours with alternative phenotypes (n = 396).

Comparing results for both Bcl-2 and p53, a significant reciprocal pattern of expression was seen (table 3, p = 0.023). Combining the results for these two markers, a total of 112/432 tumours (25.9%) displayed the p53(-) / Bcl-2(+) phenotype. A trend was noted toward a higher prevalence of this phenotype in earlier stage tumours, with a prevalence of 33.9%, 26.3%, 24.1% and 14.3% in TNM stage I-IV tumours respectively (p = 0.078). Additional strong correlations were noted between the p53(-) / Bcl-2(+) phenotype and DSS on univariate analysis (p = 0.004), and on Kaplan-Meier analysis, with a mean DSS of 84 (95%CI 75–92) months in patients with p53(-) / Bcl-2(+) tumours compared with a mean survival of 65 (95% CI 59–71) months in the remaining patients (figure 4) (p = 0.0032).

**Table 3:** Co-expression of p53 with Bcl-2.

	Number (%) p53 (+)	Number (%) p53 (-)
Number (%) Bcl-2 (+)	129 (29.8)	106 (24.5)
Number (%) Bcl-2 (-)	87 (20.1)	111 (25.6)

### Multivariate analysis

#### HRs for individual markers

A multivariate analysis of factors influencing survival was performed using the Cox proportional hazards model. Analysis included all available cases. Of the conventional clinico-pathological variables analysed, significant independent prognostic value was demonstrated only for TNM stage, and for vascular invasion status. In contrast, neither p53 expression, nor Bcl-2 expression alone were found to be independent markers of prognosis. Considering the combined p53(-) / Bcl-2(+) phenotype, this was found to have independent prognostic value, conferring a significant survival advantage as compared with the other possible combinations of p53 / Bcl-2 expression (table 4).

**Table 4:** Cox multivariate analysis.

Variable	Category	OR	95% CI	*p *value
				
TNM stage	I	1		<0.001
	II	1.427	0.758–2.689	
	III	3.170	1.728–5.816	
	IV	22.070	11.309–43.068	
	Unknown	2.895	0.640–13.102	
Vascular invasion status	Negative	1		<0.001
	Positive	2.156	1.525–3.047	
	Unknown	1.221	0.798–1.869	
				
p53/Bcl-2 status	Non- p53(-) / Bcl-2 (+)	1		0.029
	P53(-) / Bcl-2 (+)	0.659	0.452–0.959	

## Discussion

Previous studies have suggested that combined p53 and Bcl-2 immunostaining may provide useful prognostic information in colorectal cancer; however these conclusions have been derived from varying study populations, with heterogeneous methodology and limited sample sizes. To our knowledge no previous study has investigated these findings in such a large, representative cohort of patients from the UK. Using widely validated antibodies and established techniques, our results indicate that patient stratification by combined p53/Bcl-2 phenotype provides tumour stage-independent prognostic information; specifically, that a subset of up to a quarter of colorectal cancer patients display a good prognosis p53(-)/Bcl-2(+) phenotype.

In line with previous studies, we found no positive correlations between expression of the individual markers and our clinico-pathological data [18]. However, as has been the case previously [19], we noted significant reciprocity of p53 and Bcl-2 expression. Saleh *et al*, reported an association between Bcl-2 expression and lower p53 levels, a lower mean Ki-67 labelling index and favourable histopathological parameters [20]. Similarly, Popescu *et al*, demonstrated reciprocal expression of Bcl-2 and p53 mRNA in samples from 19 colorectal cancer metastases [21], however, other studies have failed to confirm this finding [22]. Although not statistically significant, we also noted a trend towards a higher prevalence of the good prognosis p53(-)/Bcl-2(+) subgroup in earlier stage tumours, in agreement with the findings of Gouissa *et al*, who demonstrated a trend for the p53(-)/Bcl-2(+) phenotype to be associated with lymph node negative tumours in a study of 108 colorectal adenocarcinomas [22].

Two recent papers have systematically reviewed the evidence for a prognostic role of p53 expression in colorectal cancer and illustrate some of the difficulties in interpreting the available literature. In the first, Anwar *et al*, described 35 studies (24 using immunohistochemical methods) in which p53 was found to be predictive of a worse outcome and 24 papers (16 employing IHC) found no association. In total only 4 of these studies included a comparable number of patients to ours, with 39 studies reporting on <150 cases [23]. In the second review Munro *et al*, included 168 articles, reporting survival outcomes on a pool of over 18 000 patients. In this large review factors such as sources of heterogeneity between studies, the effect of publication bias and various assumptions concerning the techniques used for assessing p53 abnormalities led them to limit their conclusion to the fact that in patients with a better underlying prognosis, abnormal p53 has an adverse effect on outcome [15].

It appears logical that combined analysis of p53 with Bcl-2 should prove greater than the sum of its parts, as the p53 protein is known to regulate apoptosis via the Bcl-2/BAX pathway. Native p53 inhibits Bcl-2 gene expression by transcriptional activation of the pro-apoptotic gene BAX [24]. Bcl-2 itself is a proto-oncogene, whose expression varies in differing tissues. In the normal gastrointestinal tract Bcl-2 expression has been detected in the basal cells of crypts. Bcl-2 expression has also been described in the majority (up to 85%) of colorectal adenomas, with reduced expression on progression to invasive carcinoma [25,26]. Despite the defined role of Bcl-2 protein in suppression of apoptosis, in colorectal cancer the evidence suggests that Bcl-2 expression is correlated with favourable parameters [27] and a better prognosis [28]. This may be explained by the finding that although Bcl-2 inhibits apoptosis it may also slow cell growth [29]. It has been reported that Bcl-2 contains an anti-proliferative domain, distinct from the domains required for its anti-apoptotic activity [30], and also that Bcl-2 can be converted to a BAX-like death effector by cleavage of a regulatory loop domain by caspases [31]. Whatever the underlying biological mechanism, in this study we have found that the combination of positive cytoplasmic Bcl-2 expression and negative nuclear p53 expression in colorectal cancer defines a population of patients with a good prognosis, indicating a clinically more indolent phenotype and a subset of patients for whom less aggressive adjuvant treatment is indicated.

## Conclusion

The use of molecular prognostic markers which complement traditional clinico-pathological staging information is not yet widespread in colorectal cancer, mainly due to the lack of high quality prospective studies and concerns regarding reproducibility and generalisation from existing data [32]. In particular, quantitative scoring of immunostaining for practical/diagnostic purposes is a very difficult affair and liable to much subjectivity. This is probably the major reason why so few quantitative immunomarkers have made their way into surgical pathology. These issues need to be addressed if we are to improve on existing prognostic criteria. However, our results suggest that analysis of p53 and Bcl-2 expression in colorectal cancer patients may provide useful prognostic information.

## Competing interests

The author(s) declare that they have no competing interests.

## Authors' contributions

**LGD**, **IS**, **JHS **and **IOE **conceived and designed the study.

**JHS **was responsible for maintaining the clinical database.

**NFSW**, **ZM **and **DS **were responsible for constructing the tissue arrays, performing the immunohistochemistry and analyzing the results.

All authors were responsible for interpreting the results and drafting the article. All authors read and approved the final manuscript.
